# Prevalence and spectrum of homologous recombination repair mutations in patients with metastatic prostate cancer from India

**DOI:** 10.1093/oncolo/oyag059

**Published:** 2026-02-23

**Authors:** Hemavathi Baskarane, Rishabh Jain, Mohit Kumar Divakar, Amlesh Seth, Brusabhanu Nayak, Sameer Bakhshi, Ranjit Kumar Sahoo, Akash Kumar, Aparna Sharma, Seema Kaushal, Kunhi Parambath Haresh, Vishakha Hooda, Payal Vasudeva, Pranav Pratap Singh, Rishika Agarwal, Sanskriti Vasundhara, Neeraj Agarwal, Atul Batra

**Affiliations:** Department of Medical Oncology, Dr. BR Ambedkar Institute Rotary Cancer Hospital, All India Institute of Medical Sciences, New Delhi, India; Department of Medical Oncology, Dr. BR Ambedkar Institute Rotary Cancer Hospital, All India Institute of Medical Sciences, New Delhi, India; Department of Medical Oncology, Dr. BR Ambedkar Institute Rotary Cancer Hospital, All India Institute of Medical Sciences, New Delhi, India; Department of Urology, All India Institute of Medical Science, New Delhi, 110029, India; Department of Urology, All India Institute of Medical Science, New Delhi, 110029, India; Department of Medical Oncology, Dr. BR Ambedkar Institute Rotary Cancer Hospital, All India Institute of Medical Sciences, New Delhi, India; Department of Medical Oncology, Dr. BR Ambedkar Institute Rotary Cancer Hospital, All India Institute of Medical Sciences, New Delhi, India; Department of Medical Oncology, Dr. BR Ambedkar Institute Rotary Cancer Hospital, All India Institute of Medical Sciences, New Delhi, India; Department of Medical Oncology, Dr. BR Ambedkar Institute Rotary Cancer Hospital, All India Institute of Medical Sciences, New Delhi, India; Department of Pathology, All India Institute of Medical Sciences, New Delhi, India; Department of Radiation Oncology, Dr. BR Ambedkar Institute Rotary Cancer Hospital, All India Institute of Medical Sciences, New Delhi, 110029, India; Department of Medical Oncology, Dr. BR Ambedkar Institute Rotary Cancer Hospital, All India Institute of Medical Sciences, New Delhi, India; Department of Medical Oncology, Dr. BR Ambedkar Institute Rotary Cancer Hospital, All India Institute of Medical Sciences, New Delhi, India; Department of Medical Oncology, Dr. BR Ambedkar Institute Rotary Cancer Hospital, All India Institute of Medical Sciences, New Delhi, India; Department of Medical Oncology, Dr. BR Ambedkar Institute Rotary Cancer Hospital, All India Institute of Medical Sciences, New Delhi, India; University College of Medical Sciences, New Delhi, 110095, India; Division of Medical Oncology, Department of Internal Medicine, Huntsman Cancer Institute, University of Utah, Salt Lake City, UT, 84112, United States; Department of Medical Oncology, Dr. BR Ambedkar Institute Rotary Cancer Hospital, All India Institute of Medical Sciences, New Delhi, India

**Keywords:** homologous recombination repair, somatic mutations, metastatic prostate cancer, pathogenic variant, Indian population, genomic profiling, *ATM*

## Abstract

**Background:**

Alterations in genes involved in homologous recombination repair (HRR) occur in approximately 20%-25% of patients with metastatic prostate cancer and are associated with aggressive biology, poor outcomes, and potential sensitivity to poly (ADP-ribose) polymerase inhibitors (PARPi). However, frequency and variations in somatic HRR mutations in the Indian population are unknown.

**Methods:**

We analyzed somatic HRR alterations in patients at the All India Institute of Medical Sciences, New Delhi, between 2022 and November 2024. Targeted next-generation sequencing of 15 HRR genes was performed on tumor tissue samples. Demographic and clinicopathological variables were retrieved from medical records, and their associations were assessed.

**Results:**

Of 247 patients tested, 167 were evaluable (32.3% tissue failure due to poor DNA yield). Sixty-eight pathogenic HRR alterations were detected across 51 patients (30.5%). *ATM* was the most frequently altered gene (13.2%), followed by *BRCA1* (5.3%), *BRCA2* (4.2%), and *CDK12* (4.2%). Variants of unknown significance (VUS) were detected in 12% (20) of patients. Patients with HRR alterations had higher baseline PSA values compared with the non-HRR cohort (median 150 vs 100 ng/mL, *P* = .012). No significant associations were observed with age, Gleason score, disease volume or risk category, or visceral metastases.

**Conclusions:**

This study provides the first comprehensive dataset on the spectrum of somatic HRR mutations in Indian patients with prostate cancer. The prevalence (30.5%) was somewhat higher than the global studies, *ATM* was the most frequently mutated gene, followed by *BRCA1*, in contrast to Western and Asian cohorts, where *BRCA2* predominates. These findings suggest potential population-specific variations and underscore the need for broader HRR testing to better delineate the genomic landscape of prostate cancer in Indian patients.

Implications for Practice This study provides the first comprehensive characterization of somatic HRR gene alterations in Indian men with metastatic prostate cancer. HRR alterations were identified in 30.5%, a frequency comparable to global datasets but distinctly higher than in other Asian cohorts, with a distinct gene distribution. *ATM* was the most commonly altered gene as opposed to BRCA2 predominance in other populations. These findings have significant implications for precision oncology, patient counseling, and biomarker-driven treatment for Indian and South Asian populations worldwide.

## Introduction

Prostate cancer is a significant worldwide health burden, with an estimated 1.47 million new cases diagnosed each year, and around 397 000 deaths, ranking fifth among cancer causes of death in men.^1^ In India, prostate cancer is the sixth most common cancer, with approximately 38 000 new cases diagnosed and 18 000 deaths reported annually.^1^^,^^2^ Despite advances in treatment, all patients with metastatic prostate cancer will progress to castration-resistant prostate cancer (CRPC) eventually, which carries a poor prognosis.^3^^,^^4^

Approximately 20%-25% of cases of mCRPC harbor somatic or germline HRR gene mutations.^5^^,^^6^ Tumors harboring homologous recombination repair (HRR) alterations are associated with aggressive disease, higher risk of recurrence and metastasis, and reduced survival.^7–10^ Homologous recombination repair pathway deficiencies create a “synthetic lethality” environment for Poly (ADP-ribose) polymerase inhibitors (PARPi).^11^ Clinical trials have demonstrated improved outcomes with PARPi in HRR-deficient mCRPC.^6^^,^^12^ Niraparib, olaparib and talazoparib in combination with androgen receptor pathway inhibitors (ARPIs) are approved for treatment of both germline and somatic HRR-mutated mCRPC.^5^^,^^13–18^ Accordingly, the current ASCO guidelines recommend germline genetic testing in all metastatic patients and somatic HRR testing in mCRPC to identify candidates for PARP inhibitor therapy.^12^ Both germline and somatic testing identify HRR alterations guiding PARP inhibitor therapy, with germline testing additionally informing hereditary risk and family counseling, and somatic testing capturing prognostic non-HRR alterations (e.g., TP53, RB1, SPOP, PTEN).^12^ Testing can be performed using validated NGS assays, from targeted HRR panels to whole-exome sequencing for germline, and tumor NGS, comprehensive genomic profiling, or liquid biopsy for somatic analysis.^11^^,^^12^ Homologous recombination repair panels varied across studies, and the prototype phase III PROfound trial used prospective tissue testing with a 15-gene HRR panel. Recently, the AMPLITUDE trial has demonstrated that combining niraparib with abiraterone significantly reduced the risk of radiographic progression or death by 37% in the HRR-mutated population (HR 0.63; 95% CI, 0.49-0.80; *P* = .0001).^13^ Ongoing trials, such as EVOPAR Prostate01 and TALAPRO-3, are also evaluating the role of other PARPi in upfront metastatic castration-sensitive disease, highlighting the expanding therapeutic relevance of somatic testing.^14^^,^^15^

Ethnic and geographical diversity influences the landscape of HRR mutations.^16^^,^^17^ While our center previously reported germline HRR mutation frequencies in metastatic prostate cancer with BRCA2 as the most frequently altered gene in a cohort of 276 Indian patients comparable to global studies, Asian prostate cancer studies have shown variation in mutation prevalence and distribution.^18–20^ Importantly, somatic HRR mutation data from India remain limited. To address this knowledge gap, we performed a retrospective analysis of somatic HRR alterations in an Indian prostate cancer cohort.

## Methodology

### Patients

From 2022 to November 2024, patients were screened for HRR gene mutations when clinically indicated, or as a part of a clinical trial evaluating the role of carboplatin in patients with metastatic castration resistant prostate cancer harbouring an HRR mutation.^21^

This study was conducted at the All India Institute of Medical Sciences (AIIMS), New Delhi, where we treat approximately 400-500 patients annually. The Institutional Ethics Committee approved the study.

### Demographic and clinical variables

For the current analysis, demographic and clinical details were extracted from patients’ medical records. Data collected included age at diagnosis, baseline prostate-specific antigen (PSA), and pathological characteristics such as Gleason grade and group. The extent and distribution of metastases were also documented, with disease volume and risk classification based on CHAARTED and LATITUDE trials.^22^^,^^23^ These variables were subsequently analyzed for their association with HRR mutation status.

### Sample preparation and sequencing

Tissue samples from each patient underwent targeted gene sequencing to detect somatic homologous recombination repair (HRR) variants. The HRR panel comprised of 15 genes: *BRCA1, BRCA2, ATM, BRIP1, BARD1, CDK12, CHEK1, CHEK2, FANCL, PALB2, PPP2R2A, RAD51B, RAD51C, RAD51D*, and *RAD54L*. The 15-gene HRR panel was chosen based on the PROfound trial, as olaparib is the most widely available and cost-effective PARP inhibitor in India due to the availability of generic formulation.^24^ Library preparation was performed through target gene capture using a custom capture kit. Prepared libraries were further sequenced on the Illumina sequencing platform at a 250× target depth. Alignment of sequencing data was done using the BWA aligner in the Sentieon pipeline. Next, variant identification was done through the LoFreq somatic variant caller. Intronic and synonymous variants were filtered out to retain only the non-synonymous and splice site variants for clinical interpretation. Variant annotation, including gene-based annotation and clinical annotation, was done through the VariMAT pipeline. Variants were further classified for their clinical significance as per the Association of Molecular Pathologists (AMP)/American Society of Clinical Oncology (ACSO)/College of American Pathologists (CAP) classification recommendations.^25^

### Statistical analysis

Categorical and continuous variables extracted from demographic and clinical data were described using frequencies and central tendencies. Similarly, absolute counts and percentages were used to describe variant frequency. Associations between clinical characteristics and the presence of pathogenic alterations were determined using the chi-square test and the Mann-Whitney test for continuous variables. A multivariable logistic regression model was used to assess independent associations between HRR mutation status and clinical variables. All analyses were performed using SPSS v29 (IBM, New York, United States). Each gene’s distributions of variant frequencies were visualized using a lollipop plot supported by a mutation plotter (cBioPortal, Center for Molecular Oncology at Memorial Sloan Kettering).^26^

## Results

### Baseline characteristics

Among the 247 patients who underwent sequencing, 80 tissue samples (32.3%) failed due to poor DNA yield (Figure S1). The median age at diagnosis was 65 years (interquartile range [IQR 60-72]). The median PSA level at diagnosis was 100 ng/mL (IQR 31-394). The majority (69.5%) of biopsy specimens had a Gleason score ≥8. According to the risk and volume definition used in the CHAARTED and LATITUDE trials, 58.1% of patients had high-risk and 61.1% had high-volume disease.^17^^,^^26^ Visceral metastases were detected in 49 patients (29.3%), including liver metastasis in 10 (6%). At the time of testing, 77 patients (46.1.%) had metastatic CRPC, while 62 (37.1%) had metastatic castration-sensitive prostate cancer (CSPC). A family history of cancer was documented in 17 patients (10.2%) (Table 1).

**Table 1:** Baseline characteristics.

Characteristics	Total cohort (*n* = 247)	Evaluable cohort (*n* = 167)
**Age (*n* and %), years**	66 (60–71.5)	65 (60–72)
**Median (interquartile range)**		
** <50**	12 (4.9)	7 (4.2)
** ≥50**	230 (93.1)	160 (95.8)
** NA**	5 (2)	—
**Baseline PSA (ng/nL) (median and IQR)**	119.5 (29.7–394)	100 (31–394)
**Family H/O**		
** Present**	23 (9.3)	17 (10.2)
** Absent**	166 (67.2)	113 (67.7)
** NA**	58 (23.5)	37 (22.2)
**ECOG PS**		
** 0-2**	50 (20.2)	30 (18)
** NA**	76 (30.8)	45 (26.9)
**Gleason score (*n* and %)**		
** <8**	45 (18.2)	30 (18)
** ≥8**	167 (67.6)	116 (69.5)
** NA**	35 (14.2)	21 (12.6)
**Risk** ^b^ **(*n* and %)**		
** High**	147 (57.9%)	97 (58.1)
** Low**	62 (25.1)	47 (28.1)
** NA**	38 (15.4)	23 (13.8)
**Volume** ^a^ **(*n* and %)**		
** High**	150 (59.5%)	102 (61.1)
** Low**	59 (60.7)	42 (25.1)
** NA**	38 (15.4)	23 (13.8)
**Visceral metastasis**	69 (27.9)	49 (29.3)
**Liver metastasis (*n* and %)**	12 (4.9%)	10 (6)
**Time of testing**		
** CRPC**	106 (42.9%)	7 7 (46.1)
** CSPC**	98 (39.6)	62 (37.1)
** Status not known**	43 (17.4)	28 (16.8)
**Tissue testing unsuccessful**	80 (32.3%)	—
**CSPC**	36 (45%)	
**CRPC**	29 (36%)	

Abbreviations: CRPC, Castration-resistant prostate cancer; CSPC, Castration-sensitive prostate cancer; IQR, InterQuartile Range; NA, Not Available; PSA, Prostate Specific Antigen.

a CHAARTED high-volume disease was defined as the presence of visceral metastasis or ≥4 bone lesions with ≥1 beyond the vertebral bodies and pelvis.

b LATITUDE high risk was defined as meeting at least two of the following three criteria: (1) Gleason score ≥8, (2) presence of ≥3 lesions on bone scan, and (3) presence of measurable visceral lesions.

### Variants detected

**Figure 1:**
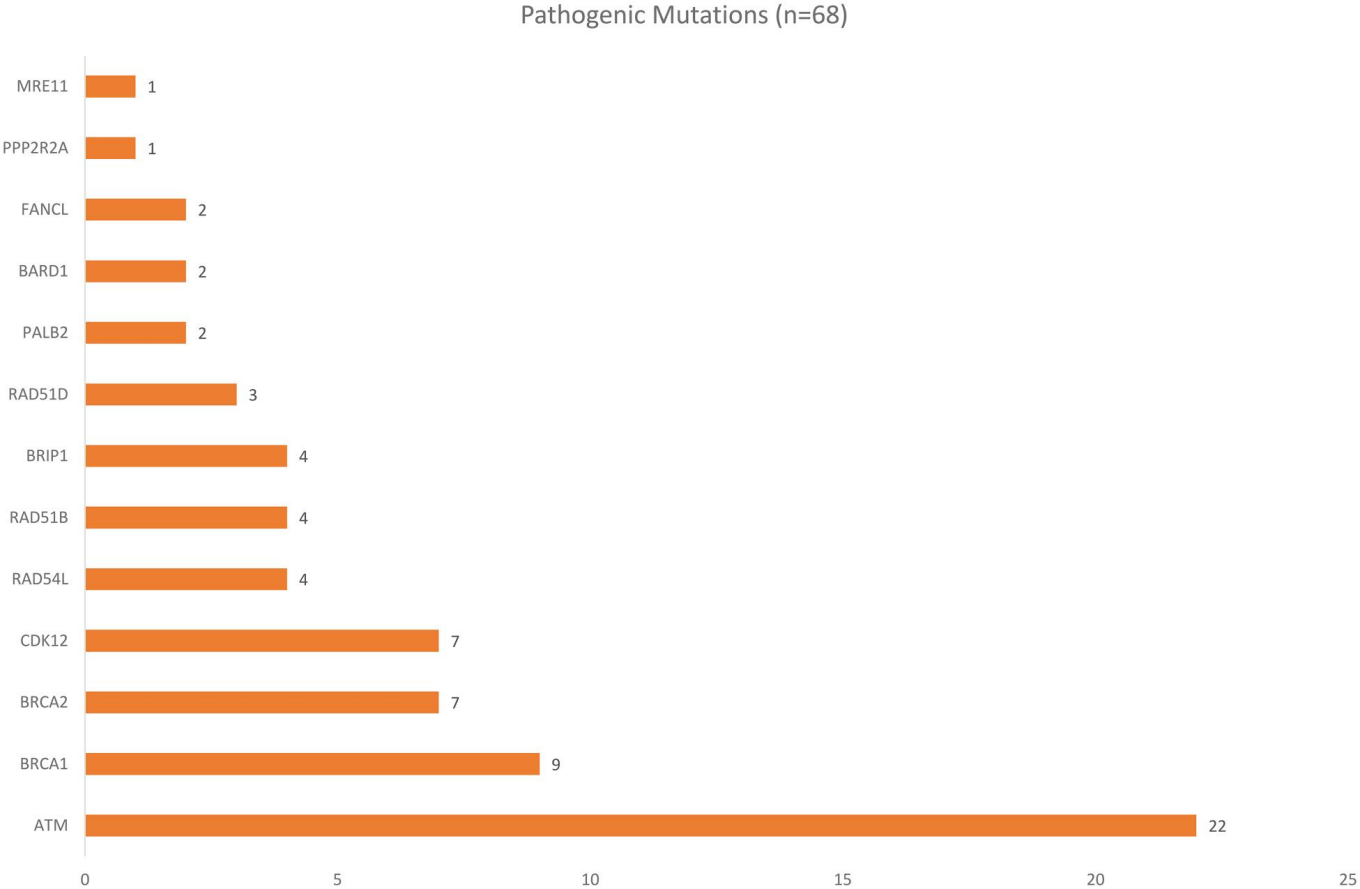
Distribution of somatic homologous recombination repair (HRR) mutations detected across 51 patients.

**Figure 2:**
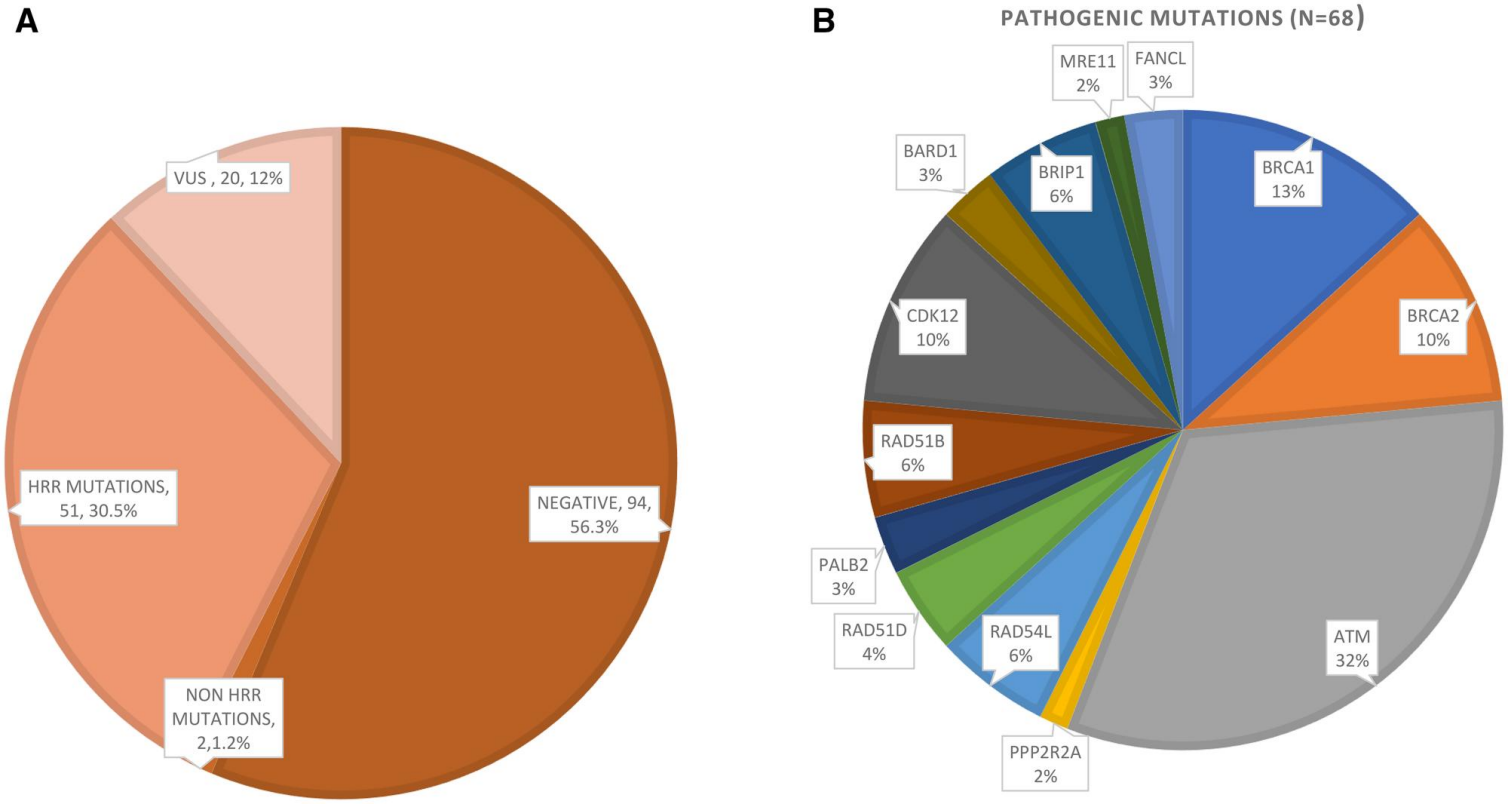
(A) Sequencing status distribution in the cohort (*n* = 167). (B) Frequency of homologous recombination repair (HRR) gene alterations detected across 51 patients.

Of the 167 evaluable samples, alteration of at least one of the HRR genes was observed in 51 patients (30.5%). In addition, 2 patients had *TP53* mutations (Figure 1). Variants of uncertain significance (VUS) were identified in 20 patients (12%) (Figure 2).

**Figure 3:**
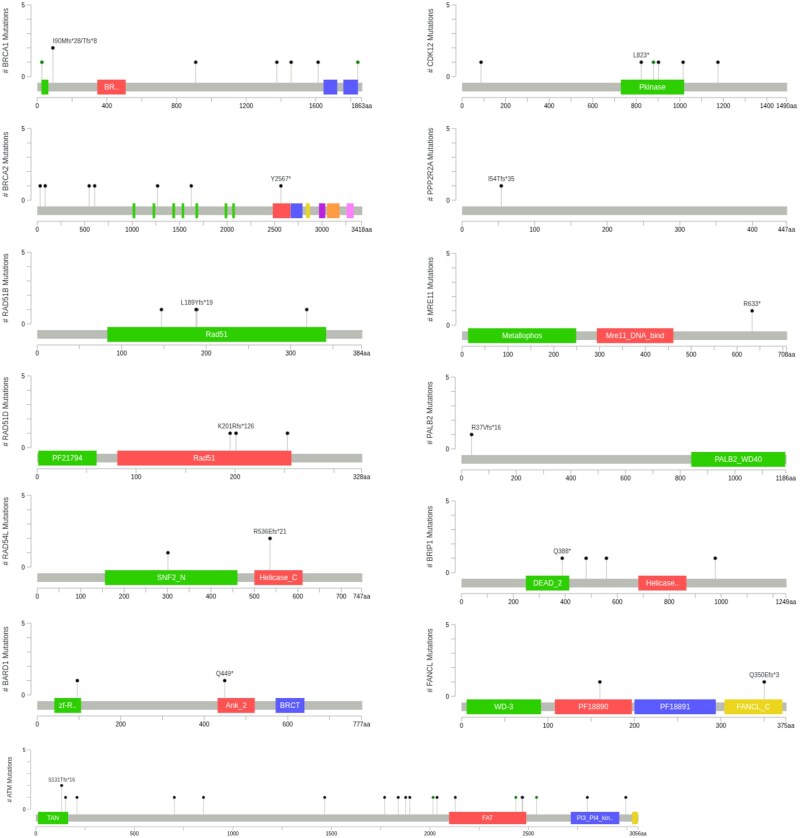
Lollipop diagrams reflecting distribution of specific variants of each gene across their respective protein sequences. Functional protein domains are highlighted by colored boxes (cBioPortal).

A total of 68 HRR pathogenic alterations were detected across these 51 patients (Figure 1, Table S1). Three most frequent alterations involved were *ATM (*22 mutations, 13.2%), followed by *BRCA1* (9, 5.3%), *BRCA2* (7, 4.2%), and *CDK12* (7, 4.2%). Other observed alterations included *RAD54L, RAD51B, and BRIP1* (4 each, 2.4%), *RAD51D* (3, 1.8%), and *PALB2, BARD1, and FANCL* (2 each, ∼1.2%). Rare alterations were seen in *PPP2R2A* and *MRE11 (*each ∼0.6%) (Figure 1). Variants were mapped to analogous amino acid positions along respective protein sequences (Figure 3). Table 2 summarizes HRR mutation prevalence and the most frequently altered genes, compared with global cohorts.

**Table 2:** Frequency distribution of HRR mutations across different cohorts.

Gene	Our study	Global cohort (profound trial)	Japanese cohort	Chinese cohort
	*n* = 167	***n* = 4426 (29)** ^a^	*n* = 143 (20)^a^	*n* = 200 (27)^a^
**HRR prevalence**	30.5%	27.9%	35.7%	3%
***BRCA2***	4.2%	9%	12.6%	0.5%
***BRCA1***	5.3%	1%	0.6%	0%
***ATM***	13.2%	6%	5.6%	1%
***CDK12***	4.2%	7%	13.3%	1%

a Numbers in parentheses indicate the corresponding reference.

Among the 51 patients with HRR mutations, 36 (21.5%) had one pathogenic alteration, 11 (6.5%) had two, and 4 (2.3%) had three (Table S2). Within the various somatic HRR mutations, we observed 20 biallelic mutations in 10 different patients; 8 biallelic alterations in *ATM* gene in 4 patients, 4 biallelic alterations in CDK12 gene in 2 patients, 4 biallelic alterations in BRCA1 gene in 2 patients, 2 biallelic alterations in BRCA2 gene in 1 patient and 2 biallelic alterations in RAD51B gene in 1 patient. On the other side, 48 monoallelic mutations were detected in 13 different genes. Interestingly, biallelic variants had a different spectrum than the mono-allelic variants; such that the same gene had different genomic alterations in biallelic form than the alleles present in monoallelic state.

### Association between patient characteristics and variants

Among the clinical parameters assessed, patients with HRR alterations had significantly higher baseline PSA levels (median 150 ng/mL [IQR 43-530]) compared with the HRR-negative cohort (median 100 ng/mL [IQR 33.7-291]) (*P* = .012). Median age was similar between the two groups (64 years [IQR 59-71] vs 66 years [IQR 60-72]; *P* = .10) (Table 3). A trend toward higher Gleason score and high-volume, high-risk disease was found among patients with HRR alterations, although this did not reach statistical significance. No significant associations were observed with family history or visceral metastases. In multivariable analysis, age (*P* = .069), disease volume (*P* = .88), or risk category (*P* = .50) similarly showed no independent association with HRR mutation status.

**Table 3:** Comparison of patients' characteristics with variant status (excluding tissue test failures).

Characteristics	HRR altered (*n* = 51)	No variant cohort (*n* = 114)	*P*-value
**Age (*n* and %) Median (IQR)**	64 (59-71)	66 (60-72)	.10
** <50**	1 (1.9)	6 (5.2)	
** ≥50**	50 (98.1)	108 (94.7)	.33
**Baseline PSA (ng/mL) (median and IQR)**	150 (43-530)	100 (33.7-291)	.012
**Family history (*n*)**	5 (9.8)	11 (9.6)	.96
**Gleason Score (*n* and %)**			
** <8**	9 (17.6)	21 (18.4)	.86
** ≥8**	36 (70.5)	78 (68.4)	
** NA**	6 (11.7)	15 (13.1)	
**Risk (*n* and %)**			
** High**	30 (58.8)	65 (57)	.076
** Low**	10 (19.6)	37 (32.4)	
** Not known**	11 (21.5)	12 (10.5)	
**Volume (*n* and %)**			
** High**	31 (60.7)	69 (60.5)	.08
** Low**	9 (17.6)	33 (28.9)	
** Not known**	11 (21.5)	12 (10.5)	
**Visceral metastasis**	17 (33.3)	32 (62.7)	.26
**Liver metastasis (*n* and %)**	4 (7.8)	6 (5.3)	.33

Abbreviations: IQR, interquartile range; NA, not available; PSA, prostate specific antigen.

Exploratory subgroup analyses showed no clinical differences in *BRCA1/2* (*n* = 13) or *ATM* mutated patients (*n* = 19) (Tables S3 and S4). In contrast, patients with non-*BRCA* HRR mutations (*n* = 38) were significantly younger (median 64 vs 67, *P* = .049) and had higher baseline PSA values (median 148 vs 100 ng/mL, *P* = .038). No additional clinicopathologic associations were identified to be different across subgroups (Table S5).

## Discussion

India comprises approximately 18% of the global population. Furthermore, patients of Indian origin represent a significant proportion of individuals with prostate cancer treated in oncology clinics worldwide. To our knowledge, this is the first study to comprehensively characterize the prevalence and spectrum of HRR gene mutations in the Indian population with metastatic prostate cancer. Among the 167 patients tested, pathogenic mutations in HRR genes were identified in 51 patients (30.5%), a frequency comparable to global estimates (11-30%) but distinctly higher than reported in Asian studies (Chinese, 3%-6%).^19^^,^^27^ However, the distribution of individual gene mutations in our study differed notably from global trends.

*ATM* was the most frequently mutated gene in our cohort (13.2%) followed by *BRCA1* (5.3%), *BRCA2* (4.2%), and *CDK12* (4.2%). This contrasts with the global and Asian datasets, where *BRCA2* is consistently reported as the most commonly altered HRR gene, occurring in 8%-12% of patients. For instance, PROFOUND observed *BRCA2* at 9% followed by *CDK12* at 7% and *ATM* at 6%, while the AACR GENIE data projected a similar pattern with *BRCA2* at 3.2%, *ATM* at 2.5%, and CDK12 at 4.3%.^28^^,^^29^ The ZENSHIN study from Japan and the CAPTURE study (Europe) similarly reported *BRCA2* at 12.6 and 9.8 (*BRCA1&2),* respectively, with *CDK12* being the frequently altered gene in the Japanese cohort (13.3%), a trend also observed in the Chinese cohort.^10^^,^^19^^,^^20^^,^^27^^,^^30^ Notably, the *BRCA1* mutation detection rate was higher than *BRCA2* in our cohort. By contrast, in our Indian cohort, *ATM* surpassed *CDK12,* and *BRCA1* was more frequent than *BRCA2*, a pattern not observed in Western or Asian populations (Table S6). These observations may probably suggest population-specific genomic variation. In our germline prostate study, mutation prevalence again mirrored global trends, with *BRCA2* being the most frequently altered gene (3.98%), while *ATM* was also commonly mutated but did not exceed *BRCA2* (2.98%).^18^

Shui et al. made a comparative analysis of two clinical genomic databases to assess variability in clinical and demographic features, noting racial differences in mutation prevalence between White and Black cohorts, but not within the Asian subset.^29^ In the GENIE database overall HRR mutation prevalence was similar across racial groups, but specific differences in distribution were observed: White patients had higher rates of *BRCA* (3.7%) and *ATM* (2.7%) mutations compared with Black patients (2.2% and 1.1%, respectively), whereas Black patients had a higher prevalence of *CDK12* mutations (6.9% vs 3.9%). Nonetheless, the representation of the Asian population within both datasets was less than 5%, precluding generalizability. Furthermore, our cohort exhibited a high rate of VUS variants (12%), emphasizing the need for routine testing and the development of region-specific variant interpretation databases to better characterize the Indian genomic landscape.

Clinically, a higher median PSA value at baseline was more frequent among patients with HRR mutations, although no other clinical features, including age, risk, disease volume, and Gleason grade, showed significant associations. In an exploratory subgroup analysis, the non-*BRCA* HRR-mutated cohort was slightly younger (median 64 vs 67 years, *P* = .049) and had a higher median PSA Value (median 148 vs 100 ng/mL, *P* = .038). No additional clinicopathologic differences were observed across other subgroups. Although HRR mutations are linked with aggressive disease biology, our study, in line with existing literature, found no definitive clinical correlates of mutation status.

Therapeutically, not all HRR mutations are predictably responsive to PARP inhibitors. Whereas patients with *BRCA1/2* alterations have demonstrated consistent clinical benefit, tumors harboring *CDK12* and *ATM* alterations have shown limited efficacy in major trials, including TRITON 3, PROPEL, and PROfound, although these subgroup analyses were limited by small sample sizes.^24^^,^^31^^,^^32^ Notably, in the AMPLITUDE trial in CSPC, efficacy testing in the HRR effector subgroup excluded *ATM* from the panel.^13^ In our cohort, *ATM* alterations were more frequent than globally reported, underscoring the need for focused evaluation of PARP inhibitor efficacy in this subgroup to better inform therapeutic decision-making.

The major limitations of our study include a 32.4% tissue failure rate due to poor DNA yield from archival formalin-fixed paraffin-embedded tissue and lack of sequential testing through repeat biopsy or ctDNA. However, the failure rate is comparable to previous reports including the Profound trial.^28^^,^^33^^,^^34^ The interval between biopsy and molecular testing is a key determinant of quality control failure, with tissue older than five years associated with significantly reduced NGS success.^33^^,^^34^ Repeat testing is seldom practiced in prostate cancer from the metastatic site since bone is the most common site, bone biopsy is challenging, and DNA yield is suboptimal due to acid based decalcification protocols that damage DNA integrity and molecular assay performance.^35^^,^^36^ Strategies to reduce failure rates include earlier somatic testing using primary tumor specimens, optimization of tissue fixation and storage, preferential sampling of soft-tissue metastases when feasible, and the use of gentler decalcification methods such as EDTA for bone biopsies.^12^^,^^33–36^ Although ctDNA offers a less invasive and more reliable alternative, it is not cost-effective in the Indian setting. In addition, our study is limited by a modest sample size and a retrospective, descriptive design without a pre-specified hypothesis, which may restrict the generalizability of the findings. Selection bias is possible, as only patients with available tumor tissue were included, and confounding factors may have influenced observed associations between clinical characteristics and HRR mutation status. While the study focuses on somatic alterations, family history of cancer may still be under-reported due to incomplete patient records, or recall bias may limit the contextual interpretation of mutation patterns in this cohort. Furthermore, the use of a focused 15-gene HRR panel may underestimate the overall prevalence of HRR alterations; larger studies incorporating broader HRR gene panels could further refine prevalence estimates in the Indian population. Treatment outcomes were also not evaluated, which might have provided additional clinical relevance. While the adoption of routine of genetic testing in India has previously been limited by the high cost of sequencing and restricted access to targeted agents, the reduction in sequencing costs and the availability of generic Olaparib at around $200 per month, emphasize the need for routine HRR testing by clinicians, which could enable to capture genomic diversity and therapeutic relevance in the Indian population.

## Conclusion

This study provides the first real-world evidence delineating the prevalence and molecular spectrum of somatic HRR gene alterations in metastatic prostate cancer among the Indian population. While the overall frequency of HRR mutations parallels global datasets, the distribution of specific gene-level alterations demonstrates distinct patterns. These findings have significant implications for precision oncology, informing patient counseling regarding genomic testing, guiding therapeutic decision-making, and optimizing the design of biomarker-driven clinical trials for patients with prostate cancer in India and those of Indian ancestry globally.

## Supplementary Material

oyag059_Supplementary_Data

## Data Availability

All data that support the findings of this study are accessible from the corresponding author upon reasonable request.
