# Supplementary material for: Prevalence and spectrum of homologous recombination repair mutations in patients with metastatic prostate cancer from India
Source: Oncologist. 2026 Feb 23;31(4):oyag059. doi: 10.1093/oncolo/oyag059 (PMC12986755; doi:10.1093/oncolo/oyag059)
Supplement: oyag059_Supplementary_Data [file oyag059_supplementary_data.zip › SUPPLEMENT THE ONCOLOGIST.docx]

**SUPPLEMENTARY:**

**TABLE S1: VARIANT DETAILS FOR THE PATHOGENIC HRR ALTERATIONS DETECTED IN OUR COHORT**

| **Sr. No.** | **Gene** | **Nucleotide change** | **Protein change** | **Variant Allele Frequency** |
| --- | --- | --- | --- | --- |
| 1 | ATM | c.2922-1G>A | Splice variant | 12.80% |
| 2 | ATM | c.185+1_185+4delGTAT | p.? | 25.00% |
| 3 | ATM | c.8395_8396insCTTCC | p.Phe2799Serfs*9 | 10.71% |
| 4 | ATM | c.6047A>T | p.Asp2016Val | 66.67% |
| 5 | ATM | c.7621C>T | p.Leu2541Phe | 60.87% |
| 6 | ATM | c.7404_7407delAAAT | p.Glu2468Aspfs*7 | 6.12% |
| 7 | ATM | c.7411_7412insATTT | p.Ile2471Asnfs*12 | 6.12% |
| 8 | ATM | c.2106_2113delGAATAATT | p.Asn703Leufs*3 | 30.00% |
| 9 | ATM | c.7307G>A | p.Arg2436Lys | 61.40% |
| 10 | ATM | c.5308del | p.Ser1770GlnfsTe r6 | 26.90% |
| 11 | ATM | c.8979del | p.Asn2994IlefsTer12 | 25.80% |
| 12 | ATM | c.624dupA | p.Phe209Ilefs*45 | 25% |
| 13 | ATM | c.4396C>T | p.Arg1466Ter | 44.68% |
| 14 | ATM | c.6108T>A | p.Tyr2036Ter | 29.30% |
| 15 | ATM | c.6387C>G | p.Tyr2129Ter | 15.10% |
| 16 | ATM | c.5631_5635delinsA | p.Phe1877LeufsTer39 | 59.90% |
| 17 | ATM | c.391_409del | p.Ser131ThrfsTer16 | *NA* |
| 18 | ATM | c.5515C>T | p.Gln1839Ter | 6.86% |
| 19 | ATM | c.5692C>T | p.Arg1898Ter | 14.80% |
| 20 | ATM | c.391_409del | p.Ser131ThrfsTer16 | 37.10% |
| 21 | ATM | c.450_453del | p.Ser151Ter | 37.80% |
| 22 | ATM | c.2551del | p.Asp851IlefsTer5 | 62.90% |
| 23 | BARD1 | c.285_286insGAGG | p.Lys96Glufs*5 | 13.79% |
| 24 | BARD1 | c.1345C>T | p.Gln449Ter | 6.52% |
| 25 | BRCA1 | c.4125_4126insGGAAG | p.Thr1376Glyfs*19 | 12.50% |
| 26 | BRCA1 | c.268_269insCAAA | p.Ile90Thrfs*8 | 2.76% |
| 27 | BRCA1 | c.81-1G>A | Splice variant | 5.20% |
| 28 | BRCA1 | c.5522G>A | p.Ser1841Asn | 22% |
| 29 | BRCA1 | c.270_273delTTGT | p.Ile90Metfs*28 | 2.63% |
| 30 | BRCA1 | c.4372C>T | p.Gln1458Ter | 5.26% |
| 31 | BRCA1 | c.80G>A | p.Cys27Tyr | 2% |
| 32 | BRCA1 | c.4837_4838delinsGCC | p.Ser1613Ala fsTer9 | 43.70% |
| 33 | BRCA1 | c.2728C>T | p.Gln910Ter | 26.50% |
| 34 | BRCA2 | c.92G>A | p.Trp31Ter | 11.80% |
| 35 | BRCA2 | c.7701T>G | p.Tyr2567Ter | 43.80% |
| 36 | BRCA2 | c.4867C>T | p.Gln1623Ter | 47.40% |
| 37 | BRCA2 | c.3803_3804del | p.Ser1268Cysfs*6 | NA |
| 38 | BRCA2 | c.250C>T | p.Gln84Ter | 31.80% |
| 39 | BRCA2 | c.1640C>G | p.Ser547Ter | 12.90% |
| 40 | BRCA2 | c.1813delA | p.Ile605Tyrfs*9 | 33.33% |
| 41 | BRIP1 | c.2930_2939delCAGGGAAAGC | p.Ala977Glufs*5 | 25.00% |
| 42 | BRIP1 | c.1438del | p.Met480TrpfsTer46 | 13.20% |
| 43 | BRIP1 | c.1162C>T | p.Gln388Ter | 4.29% |
| 44 | BRIP1 | c.1674G>A | p.Trp558Ter | 5.10% |
| 45 | CDK12 | c.2704C>T | p.Arg902Ter | 26.20% |
| 46 | CDK12 | c.3524del | p.Pro1175GlnfsTer5 | 16.80% |
| 47 | CDK12 | c.2636G>C | p.Gly879Ala | 2.76% |
| 48 | CDK12 | c.259_260del | p.Met87GlyfsTer3 | 66.80% |
| 49 | CDK12 | c.3042_3043del | p.Leu1015Thrfs*6 | 20.50% |
| 50 | CDK12 | c.2466_2469del | p.Leu823Ter | 13.50% |
| 51 | CDK12 | c.1047-2A>G | NA | 51.50% |
| 52 | FANCL | c.1048_1049delCA | p.Gln350Glufs*3 | 12.50% |
| 53 | FANCL | c.476_477insAGAG | p.Ala160Glufs*30 | 37.80% |
| 54 | MRE11 | c.1897C>T | p.Arg633Ter | 66.70% |
| 55 | PALB2 | c.1685-1G>A | Splice Site Variant | 8.42% |
| 56 | PALB2 | c.109del | p.Arg37ValfsTer16 | 54.70% |
| 57 | PPP2R2A | c.160_161insCT | p.Ile54Thrfs*35 | 11.54% |
| 58 | RAD51B | c.439_440insC | p.Phe147Serfs*2 | 27.27% |
| 59 | RAD51B | c.953_954insGTCA | p.Gln319Serfs | 10.71% |
| 60 | RAD51B | c.561delA | p.Val188Phefs*20 | 25.00% |
| 61 | RAD51B | c.564delT | p.Leu189Tyrfs*19 | 25.00% |
| 62 | RAD51D | c.601_602insG | p.Lys201Argfs*126 | NA |
| 63 | RAD51D | c.583_590delGCTTCTTC | p.Gly195Argfs*129 | 2.63% |
| 64 | RAD51D | c.757C>T | p.Arg253Ter | 5.20% |
| 65 | RAD54L | c.1604del | p.Arg536GlufsTer21 | 35% |
| 66 | RAD54L | c.896_899dup | p.Leu301Glnfs*6 | 33.60% |
| 67 | RAD54L | c.3+3_3+4insCC | NA | 17.12% |
| 68 | RAD54L | c.1606del | p.Arg536GlufsTer21 | 51.60% |

**TABLE S2: COMBINATION OF HRR GENE MUTATIONS AND THEIR PATIENT FREQUENCIES**

|  | MUTATION COMBINATION | No of patients |
| --- | --- | --- |
| 1 | *BRCA1- 2 VARIANTS* | 2 |
| 2 | *BRCA2-2 VARIANTS* | 1 |
| 3 | *ATM-2 VARIANTS* | 3 |
| 4 | *CDK12-2 VARIANTS* | 2 |
| 5 | *BRCA 1, BRIP 1* | 1 |
| 6 | *ATM, BARD1* | 1 |
| 7 | *BRCA2, ATM* | 1 |
| 8 | *RAD 51B, BRCA 1* | 1 |
| 9 | *ATM, BARD 1, BRIP 1* | 1 |
| 10 | *CDK 12, ATM, ATM*  *CDK12, CDK12, ATM* | 1  1 |
| 11 | *RAD51B, RAD51B, FANCL* | 1 |

**TABLE S3: CORRELATION BETWEEN THE PRESENCE OF BRCA1/2 STATUS AND VARIOUS CLINICAL/PATHOLOGICAL CHARACTERISTICS**

| Characteristics | BRCA1 &2 mutation (n=13) | No mutation (n=114) | p-value |  |  |
| --- | --- | --- | --- | --- | --- |
| Age (n and %)  <50  >=50  NA | 61.5(54-68.5)  0  13(100)  - | 66(60-72)  6(5.2)  108(94.7) | 0.43  0.37 |  |  |
| Baseline PSA (ng/nl), median and IQR | 148(18.1-676) | 100(33.7-291) | 0.45 |  |  |
| Gleason Score (n and %)  <8  >=8  NA | 1(7.6)  10(76.9)  2(15.3) | 21(18.4)  78(68.4)  15(13.1) | 0.31 |  |  |
| ECOG PS  0-2  3-4  NA | 9(69.2)  1(7.7)  3(23) | 74(65)  6(5.2)  34(29.8) | 0.57 |  |  |
| Risk(n and %)  High  Low  NA | 9(69.2)  1(7.7)  3(23) | 65(57)  37(32.4)  12(10.5) | 0.14 |  |  |
| Volume (n and %)  High  Low  NA | 9(69.2)  1(7.7)  3(23) | 69(60.5)  33(28.9)  12(10.5) | 0.19 |  |  |
| Visceral mets | 6(46.1) | 32(62.7) | 0.3 |  |  |
| Liver metastasis (n and %) | 1(7.6) | 6(5.3) | 0.84 |  |  |
| Abbreviations: IQR, InterQuartile Range; NA, Not Available; PSA, Prostate Specific Antigen | | | |  |  |

**TABLE S4: CORRELATION BETWEEN THE PRESENCE OF ATM MUTATION AND VARIOUS CLINICAL/PATHOLOGICAL CHARACTERISTICS**

| Characteristics | ATM Mutation  (N=19) | No mutation  (N=114) | p-value |
| --- | --- | --- | --- |
| Age (n and %) MEDIAN(IQR)  <50  >=50 | 64 (63–66)  1(5.2)  18(94.7) | 66(60-72)  6(5.2)  108(94.7) | 0.07  0.69 |
| Baseline PSA (ng/nl), median and IQR | 130 (43–400) | 100 (33.1–300.5) | 0.31 |
| Gleason Score (n and %)  <8  >=8  NA | 4(21)  13(68)  2(10.5) | 21(18.4)  78(68.4)  15(13.1) | 0.53 |
| ECOG PS  0-2  3-4  NA | 9(47.4)  3(15.8)  7(36.8) | 74(65)  6(5.2)  34(29.8) | 0.07 |
| Risk (n and %)  High  Low  NA | 12(63)  5(26)  2(10.5) | 65(57)  37(32.4)  12(10.5) | 0.87 |
| Volume (n and %)  High  Low  NA | 12(63.1)  5(26.1)  2(10.5) | 69(60.5)  33(28.9)  12(10.5) | 0.97 |
| Visceral mets | 6(31.5) | 32(62.7) | 0.95 |
| Liver metastasis (n and %) | 1(5.2) | 6(5.3) | 0.99 |
| Abbreviations: IQR, InterQuartile Range; NA, Not Available; PSA, Prostate Specific Antigen | | | |

**TABLE S5: CORRELATION BETWEEN NON- BRCA HRR MUTATION STATUS AND VARIOUS CLINICAL/PATHOLOGICAL CHARACTERISTICS**

| Characteristics | Non-BRCA HRR altered (N=38) | No mutation (N=114) | p-value |
| --- | --- | --- | --- |
| Age (n and %) MEDIAN  <50  >=50 | 64(59-71)  1(2.6)  37(97.3) | 66(60-72)  6(5.2)  108(94.7) | 0.049/0.14  0.69 |
| Baseline PSA (ng/nl), median, and IQR | 148(52.9-566) | 100(33.7-291) | 0.038 |
| Gleason Score (n and %)  <8  >=8  NA | 8(21)  29(76.3)  3(7.8) | 21(18.4)  78(68.4)  15(13.1) | 0.96 |
| ECOG PS  0-2  3-4  NA | 22(57.9)  6(15.8)  10(26.3) | 74(65)  6(5.2)  34(29.8) | 0.74 |
| Risk (n and %)  High  Low  NA | 21(55.2)  10(26.3)  7(18.4) | 65(57)  37(32.4)  12(10.5) | 0.51 |
| Volume (n and %)  High  Low  NA | 22(57.8)  9(23.6)  7(18.4) | 69(60.5)  33(28.9)  12(10.5) | 0.52 |
| Visceral mets | 11(29) | 32(62.7) | 0.74 |
| Liver metastasis (n and %) | 3(7.8) | 6(5.3) | 0.62 |
| Abbreviations: IQR, InterQuartile Range; NA, Not Available; PSA, Prostate Specific Antigen | | | |

**TABLE S6: SUMMARY OF KEY STUDIES ON SOMATIC HRR PREVALENCE**

| Study | Study population | Genes tested | Somatic prevalence | Germline | Somatic Gene Frequency | Region |
| --- | --- | --- | --- | --- | --- | --- |
| CAPTURE  Study  Olmos et al, 2024(10) | 729  mCRPC | HRR panel (tumor ± germline) | 30% HRR;  Somatic + germline | - | Somatic *BRCA-9.8%*  Somatic *HRR non BRCA-*13.4% | Europe |
| Cross-sectional EU survey  Castro et al, 2025 (32) | 640  mCRPC | HRR Panel | 17% | 13% | Not reported | Europe |
| PROSPECT study  Manneh et al, 2024(34) | 387 | HRR panel (tumor ± germline) | Not estimable (somatic)  79% tissue failure | 4.2% germline | - | Latin America & Caribbean |
| Chehade et al,2025 (7) | 637 de novo mHSPC; | FoundationOne CDx assay  (Tumour) | 28.4% | - | *BRCA1/2 (10.8%)*  *CDK12 (7.4%) ATM (4.8%)* | US (multi-ethnic) |
| Prendeville et al, 2024(35) | 516 (localized + metastatic) | HRR (BRCA, ATM, PALB2) & MMR genes | 12.0% | - | *BRCA2 (33.3%), ATM (26.7%) BRCA1 (11.1%)*  *PALB2 (3.3)* | Canada |
| ASIAN COHORT | | | | | | |
| ZENSHIN STUDY 2022(24) | 143  mCRPC | 15 gene HRR panel | 35.7% | - | *CDK12 -13.3%), BRCA2*  *12.6%*  *ATM- 5.6% CHEK2*  *2.1%* | Japan |
| Chinese study  Jiang et al  2022(23) | 74  Localised and metastatic | 19 gene HRR panel | 6.76 % | 5.41 %, | *CDK12 -30%* | China |
| Liu et al,2023 (30) | Localised and metastatic  200-tissue  714-germline | 32 gene panel | 3% | 3.9% | *ATM-1%*  *BRCA2-0.5%*  *BRCA1-0%* | China |
| PARPI stratified trials | | | | | | |
| PROFOUND  (29) | mCRPC  N=4426 | 15 Gene panel | 27.9% | - | *BRCA2 (~9%), CDK12 (~7%), ATM (~6%), BRCA1 (~1%)* | Global |
| Genomic database | | | | | | |
| Abida et al., 2019 (MSK-IMPACT)(36) | Localised and metastatic  N=451 | MSK-IMPACT panel | 22% | - | *BRCA2-7%*  *BRCA1 –1%*  *ATM-5%*  *CDK12-7%* | US (multi-ethnic) |
| Robinson et al, 2015  SU2C-PCF(37) | 150 mCRPC | Whole-exome sequencing | 22.7 % including germline | – | *BRCA2-12.7%(germline + somatic)* | US |
| Shui et al: analysis of  2 genomic databases -  AACR GENIE + CGDB 2023(31) | mCRPC  N=3757 | CGDB – FoundationOne CDx assay | 11% -GENIE  24.6% - combined germline & somatic in CGDB | - | *BRCA2 3.2%*  *BRCA1 0.5%*  *ATM-2.5%*  *CDK12-4.3%* | US |
